# Effect of Bicycle Ergometer Training and Nordic Walking Training on Improving Functional Exercise Capacity in Asthma Patients

**DOI:** 10.7759/cureus.49762

**Published:** 2023-11-30

**Authors:** Ramachandran Sivagnanam, Ramalakshmi Krishnan, Jayabharathi Ramamoorthy, Saraswathi Karthikeyan, Srisaisantoshini Sankaranarayanan, Gayathri Kumar, Annie Janet, Selvaraj Sudhakar, Mohan Kumar Govindaraj, Veena Kirthika S

**Affiliations:** 1 Cardiovascular and Pulmonary Medicine, Faculty of Physiotherapy, Dr MGR Educational and Research Institute, Chennai, IND; 2 Sports Medicine, Faculty of Physiotherapy, Dr MGR Educational and Research Institute, Chennai, IND; 3 Orthopaedics, Faculty of Physiotherapy, Dr MGR Educational and Research Institute, Chennai, IND; 4 Neurology, Faculty of Physiotherapy, Dr MGR Educational and Research Institute, Chennai, IND

**Keywords:** functional capacity, quality of life, nordic walking, bicycle ergometer, bronchial asthma

## Abstract

Introduction

Asthma is a chronic respiratory condition characterized by inflammation of the airway leading to breathlessness. Exercise training has been recognized as a valuable component in the management of asthma, enhancing lung function and overall well-being. Bicycle ergometer training and Nordic walking are two distinct forms of exercise that have been shown to improve cardiovascular fitness and respiratory function. Despite the potential benefits of these exercises, limited research directly compares their efficacy in improving functional capacity specifically in asthma patients. The study thus aims to address this gap by providing personalized, tailored exercise programs for asthma patients.

Methods

A single-blinded experimental study using a simple random sampling method was conducted. A sample of 40 subjects was recruited for the study based on inclusion and exclusion criteria and were assigned into two groups. Group A subjects were trained with a bicycle ergometer and Group B subjects were trained with Nordic walking. The intervention was given to both groups for 12 weeks. The outcome measures used were the six-minute walk test, Modified Borg Scale, and Mini Asthma Quality of Life Questionnaire.

Results

A baseline analysis of outcome measures was done, which was followed up by a post-test analysis after 12 weeks. Pre-test and post-test data were compared using a paired t-test. Intergroup analysis was done by an independent t-test. Both groups showed significant improvement in post-test results. On comparing the two groups, Group A showed significant improvement as compared to Group B.

Conclusion

The study concludes that bicycle ergometer training is effective in improving functional capacity and enhancing the quality of life in asthma patients.

## Introduction

Asthma is characterized by chronic airway inflammation and increased tracheobronchial responsiveness to various stimuli, which poses a significant challenge globally [1]. Triggers such as allergens, infections, stressors, and environmental factors exacerbate this condition, impacting both children and adults [2]. The prevalence of asthma is about 5-10% in children and 3% in adults [3]. The consequences of bronchial asthma extend beyond the physiological aspect influencing daily life, education, work performance, and activity levels of the affected individuals. The multifaceted approach to asthma management involves medical interventions, predominantly inhalation medications, and non-medical strategies with the goal of enabling a normal lifestyle, minimizing asthma attacks, and optimizing lung function [3,4].

In the context of physical activity and asthma: Aerobic exercise can induce exercise-induced bronchoconstriction (EIB); however, regular physical activity remains crucial for overall asthma management. The fear of breathlessness often hinders asthma patients from engaging in physical activities that leads to decreased physical fitness. Hence, tailored physical training programs have been developed to enhance fitness, neuromuscular coordination, and self-confidence in individuals with asthma [5,6]. A recent international guideline regarding physiotherapeutic management of adult patients recommends breathing exercises for patients with asthma to increase asthma control and quality of life [7]. Similarly, physical training is advised to increase fitness dyspnea and improve quality of life [4].

The bicycle ergometer is commonly used as a stationary bike that has cardiovascular benefits. On a stationary bike, the upper body can stay quite steady, which helps accurately measure blood pressure [8]. Additionally, studies have shown that the use of bicycle ergometers can improve heart function and reduce breathing difficulties in asthma patients while doing moderate exercise [9]. However, it is important to note that the accuracy of this method is not always consistent because it relies on how fast the person is pedaling [10]. Nordic Walking is characterized by using poles during walking. It has gained popularity in physical therapy due to its simple and accessible characteristics for people of various ages [11]. It was developed in Scandinavia and introduced in central Europe nearly 20 years ago. People of all ages quickly were attracted by it [12]. Nordic walking proved to be a simple and feasible form of physical activity that can be done by nearly everybody, everywhere, and at almost any time [13]. Nordic walking exerts beneficial effects on resting heart rate, blood pressure, exercise capacity, maximal oxygen consumption, and quality of life in patients with various diseases and can thus be recommended to a wide range of people as primary and secondary prevention [14].

This study compares the effectiveness of two interventions - bicycle ergometer training and Nordic walking training - in improving functional exercise capacity in asthma patients. The rationale for this research lies in the potential of these interventions to enhance exercise capacity, a critical aspect of asthma management. This study aims to contribute insight into optimizing exercise interventions for asthma patients by examining the effectiveness of bicycle ergometer training versus Nordic walking training in improving their functional exercise capacity and quality of life.

## Materials and methods

Selection of subjects

This work has been conducted in the Outpatient Department of Physiotherapy in ACS Medical College and Hospital. The study design was experimental, and randomization of the individuals was done by using a simple random method. A total of 40 subjects consisting of 26 females and 14 males in the age group of 18-65 years were included in the study based on inclusion and exclusion criteria [15]. The inclusion criteria were subjects diagnosed with asthma confirmed by the physician, subjects who provided informed consent, and those willing to participate in the study. The exclusion criteria included acute exacerbation of the asthma symptoms, patients with cardiac disease, any neurological disorder, unstable vital signs, and patients with any orthopedic conditions.

Ethical considerations

The Faculty of Physiotherapy, Dr MGR Educational and Research Institute (Deemed to be University) Institutional Review Board issued approval C-23/PHYSIO/IRB/2022-2023.

Procedure

After a thorough explanation of the protocols to all the participants, they were provided with a consent form approved by the ethical committee. Then, subjects were assigned into two groups of 20 each. Group A subjects were trained with a bicycle ergometer and Group B subjects were trained with Nordic walking. The duration of the intervention was 12 weeks with a frequency of 3 days a week [16]. All 40 members in both groups were screened for pre and post-intervention assessments using outcome measures.

Intervention

Bicycle Ergometer Training: For Group A

Group A was given bicycle ergometer training for three days a week for a duration of 40 minutes per session. This includes 10 minutes of warm-up, 20 minutes of bicycle ergometer training, and the last 10 minutes of cool down. Patients were instructed to pedal at a rate of 60 revolutions per minute (RPM) without exceeding their 75% maximal heart rate (MHR). The heart rates by pulse oximetry of an individual were monitored.

Nordic Walking Training: For Group B

Group B was given Nordic walking for 3 days a week for a duration of 40 minutes per session. This includes 10 minutes of warm-up, 20 minutes of Nordic walking training, and the last 10 minutes of cool down. Each patient is instructed to start by walking naturally holding the poles on either side. Patients are asked to relax their shoulders and arms and swing from their shoulders, maintaining their upper body slightly leaned forward. Arms and legs move alternatively, that is the leading foot strikes the ground while the opposite pole strikes the ground level with the level of the leading foot. The poles always point diagonally backward and are planted between the front and back feet. Push the pole as far as possible, with the arm fully extended to form a continuous line. The palm of the hand is open and the fingers are stretched out at the release position.

Outcome measures

The outcome measures used in the study are functional capacity and quality of life. The study employs the six-minute walk test (6MWT), Borg Rating of Perceived Exertion (RPE) scale, and the Mini Asthma Quality of Life Questionnaire as evaluative tools. The 6MWT is a submaximal exercise test used to assess the functional capacity of patients with cardiopulmonary diseases [17,18]. The Borg Rating of Perceived Exertion (RPE) scale was developed by Swedish researcher Gunnar Borg. It is a tool for measuring an individual’s effort and exertion, breathlessness, and fatigue during physical work and so is highly relevant for occupational health and safety practice [19]. In its simplest terms, it provides a measure of subject experiences, including increased heart rate, increased respiration or breathing rate, increased sweating, and muscle fatigue. The Mini Asthma Quality of Life Questionnaire measures the disease-specific quality of life [20]. Asthma-related quality of life refers to the impact that asthma has on the patient’s quality of life [21].

## Results

The collected data were tabulated and analyzed using both descriptive and inferential statistics. All the parameters were assessed using Statistical Package for Social Science (SPSS) version 24 (IBM Corp., Armonk, NY, USA). A paired t-test was adopted to find the statistical difference within the groups and the independent t-test (student's t-test) was adopted to find the statistical difference between the groups.

The pre and post-test analysis of Group A and Group B asthma patients in terms of the 6MWT, Modified Borg Scale, and Mini Asthma Quality of Life Questionnaire showed a significant increase in the post-test mean values of both groups. This indicates that pretest and post-tests within Group A and Group B on asthma patients show a highly significant difference in mean values at P ≤ 0.001 (Table 1 and Table 2). On comparing the groups, Group A (bicycle ergometer training) has a higher mean value in the 6MWT, Modified Borg Scale, and Mini Asthma Quality of Life Questionnaire than Group B (Nordic walking) at P ≤ 0.001 (Table 3).

**Table 1:** Effect of bicycle ergometer training on improving functional exercise capacity in asthma patients within Group A SMWT (six-minute walk test), MBS (Modified Borg Scale), MINIAQLQ (Mini Asthma Quality of Life Questionnaire), SD (standard deviation)

GROUP A	PRE		POST		t- Value	Sign
	Mean	SD	Mean	SD		
SMWT	302	41.0	854.9	105.9	-15.38	000**
MBS	7.5	1.08	2.7	0.67	11.91	000**
MINIAQLQ	28.9	2.37	81.5	5.4	-28.18	000**

**Table 2:** Effect of Nordic walking training on improving functional exercise capacity in asthma patients within group B SMWT (six-minute walk test), MBS (Modified Borg Scale), MINIAQLQ (Mini Asthma Quality of Life Questionnaire), SD (standard deviation)

GROUP B	PRE		POST		t- Value	Sign
	Mean	SD	Mean	SD		
SMWT	327	70.09	616	54.4	-10.29	000**
MBS	7.8	0.78	5.3	0.67	7.61	000**
MINIAQLQ	30.1	4.01	59.2	5.8	-12.92	000**

**Table 3:** Effect of bicycle ergometer training versus Nordic walking training on improving functional exercise capacity in asthma patients between Group A and Group B in post-test SMWT (six-minute walk test), MBS (Modified Borg Scale), MINIAQLQ (Mini Asthma Quality of Life Questionnaire), SD (standard deviation)

POST-TEST VARIABLES	GROUP A		GROUP B		t- Value	Sign
	Mean	SD	Mean	SD		
SMWT	854.9	105.9	616	54.4	6.34	000**
MBS	2.7	0.67	5.3	0.67	-8.61	000**
MINIAQLQ	81.5	5.4	59.2	5.8	8.82	000**

## Discussion

The results of this study support the hypothesis that both bicycle ergometer training and Nordic walking training are effective in improving functional exercise capacity in asthma patients. Further analysis also supports that there is a significant difference in the effectiveness of bicycle ergometer training and Nordic walking training. Subjects treated with bicycle ergometer training demonstrated significant improvement in functional exercise capacity and quality of life. The mechanism by which bicycle ergometer training is effective is based on principles of exercise training. Exercise training is a structured intervention for patients with pulmonary diseases that has been shown to improve exercise tolerance and reduce dyspnea and quality of life [22].

The effectiveness of bicycle ergometer training is based on the principles of exercise training. Exercise training is a structured intervention for individuals with pulmonary conditions. This approach has consistently shown results in enhancing exercise tolerance, reducing dyspnea, and improving the quality of life for individuals with chronic respiratory diseases. The primary objectives of exercise training include symptom reduction, decreased disability, increased engagement in physical and social activities, and an overall enhancement of life for those with respiratory conditions [23].

Several studies have been done on exercise training in asthma patients with some studies supporting ergometer training. Pothirat C et al. (2014) found that ergometry training effectively improved peripheral and respiratory muscle strengths, dyspnea, and exercise capacity for at least nine months after the intervention [24]. Another study by Shah D et al. (2012) concluded that cycle ergometry is a preferable exercise option for individuals with asthma, showing positive effects on cardiovascular status [25]. In comparison, a singular study on Nordic walking by Prado MJ et al. (2023) suggested that combining Nordic walking with education and standard care could enhance exercise tolerance and reduce other asthma-related symptoms [26].

Asthma is a major non-communicable disease affecting both children and adults. If left untreated, it can cause sleep disturbances, tiredness during the day, poor concentration, and even a loss of quality of life. Therefore, the management and intervention for asthma should be extended to maintain functional capacity, dyspnea, and quality of life, especially with bicycle ergometers as compared to Nordic walking. The analysis of the 6MWT, Modified Borg Scale, and Mini Asthma Quality of Life Questionnaire indicates that bicycle ergometer training is more effective than Nordic walking.

Limitations of the study

The study includes small samples that cannot generalize the outcome results. Moreover, this study has been conducted on subjects in a specific setup of a particular region. The geographical variance has not been considered. The nutritional aspects of asthma patients are not included in the study.

Future scope of study

A large sample study needs to be done to know its effectiveness. The long-term effects of exercise training on the functional capacity of asthma patients could be considered in future studies.

## Conclusions

Based on the results of the present study, we conclude that there exists a positive effect of both bicycle ergometer training and Nordic walking on improving functional exercise capacity in individuals with asthma. However, a comparison reveals that bicycle ergometer training exhibits greater effectiveness in improving functional exercise capacity when compared to Nordic walking among asthmatic patients. This can contribute to more tailored and effective exercise interventions for individuals managing asthma.
